# Mendelian randomization indicates that atopic dermatitis contributes to the occurrence of diabetes

**DOI:** 10.1186/s12920-023-01575-y

**Published:** 2023-06-15

**Authors:** Feiwei Lu, Boting Wu, Yongshi Wang

**Affiliations:** 1 Department of Echocardiography, Zhongshan Hospital Fudan University, Shanghai, 200032 China; 2 Department of Transfusion, Zhongshan Hospital Fudan University, Shanghai, 200032 China; 3 Shanghai Institute of Cardiovascular Diseases, Zhongshan Hospital Fudan University, 180 Fenglin Road, Shanghai, 200032 China

**Keywords:** Mendelian randomization, Atopic dermatitis, Type 1 diabetes, Type 2 diabetes, Genome-wide association study

## Abstract

**Background:**

An association has been indicated between atopic dermatitis (AD), a prevalent chronic inflammatory skin disease, and diabetes mellitus. However, the exact causal relationship between AD and both type 1 diabetes (T1D) and type 2 diabetes (T2D) remains controversial. This study aimed to explore the causal association between AD and diabetes by Mendelian Randomization (MR) approaches.

**Methods:**

Public genetic summary data for AD was obtained from EAGLE study. Single nucleotide polymorphisms of diabetes were retrieved from four genome-wide association studies that had been performed in European populations. Inverse variance weighted (IVW) in MR analysis was used as the primary means of causality estimation. Several complementary analyses and sensitivity analyses were performed to calculate MR estimates and to enhance the causal inference, respectively. The R package ‘TwoSampleMR’ was used for analysis.

**Results:**

Genetically predicted AD led to a higher risk of T1D (OR, 1.19; 95% CI, 1.05, 1.34; *P* = 0.006) and T2D (OR, 1.07; 95% CI, 1.02, 1.11; *P* = 0.003) based on random-effect IVW method. The complementary analyses provided similar positive results. Cochran’s Q test and I^2^ statistics indicated moderate heterogeneity between AD and both T1D and T2D. No significant horizontal pleiotropy was detected by MR-Egger Intercept p except summary data from FinnGen consortium.

**Conclusion:**

Genetically predicted AD is a risk factor for both T1D and T2D. These findings imply potential shared pathological mechanisms between AD and diabetes, thus suggesting the significance of early clinical diagnosis and prevention of AD in reducing the incidence of diabetes.

**Supplementary Information:**

The online version contains supplementary material available at 10.1186/s12920-023-01575-y.

## Introduction

Diabetes is a collection of disorders characterized by impaired glucose metabolism, particularly hyperglycemia, which can cause long-term microvascular complications and non-specific macrovascular complications [1]. The latest International Diabetes Federation report shows that more than 10.5% of adults worldwide are diabetic and expected to account for 12.2% by 2045 [2]. Type 1 diabetes (T1D) is an autoimmune disease characterized by T cell-mediated destruction of pancreatic β cells and absolute deficiency of insulin [3, 4]. The risk of cardiovascular events in patients with T1D is ten times higher than in non-diabetic population [5]. High plasma concentrations of Omega-3 fatty acids in infancy and childhood vitamin D supplementation may reduce the risk of islet autoimmunity [6, 7]. Type 2 diabetes (T2D) is defined as a chronic metabolic disease featured with insulin resistance and deficiency in insulin secretion [8], which is associated with additional metabolic disorders such as dyslipidemia and atherosclerosis [9]. T2D patients carry an essential risk for cardiovascular disease (CVD) [10]. Lifestyle changes can reduce the risk of T2D [11], allowing better diabetes prevention, lower family financial burden and increased life expectancy. Recently, it has been revealed that atopic dermatitis (AD) is related to the risk of T1D and lifetime prediabetes [12, 13].

Previously called atopic eczema, AD is a complex chronic inflammatory skin disease with diverse clinical manifestations and symptoms suffered by approximately 20% of children and 3% of adults worldwide, with the incidence still increasing [14]. AD was proved to be a potential risk factor for several autoimmune diseases (OR = 1.97; 95% CI, 1.93–2.01) including T1D (OR = 1.08; 95% CI, 1.03–1.14) [15]. Wu et al. showed that the prevalence of T1D was significantly higher in patients with AD [13]. In addition, AD directly increased the risk of metabolic diseases especially T2D after adjusting for age, sex, metabolic disorders and other CVD (HR = 2.96; 95% CI, 2.56–3.41, *P* < 0.001) [16]. In multivariate models controlling for socio-demographic characteristics, smoking history, drinking history and strenuous activity, AD was still associated with a higher risk of diabetes (OR, 1.37; 95% CI, 1.16–1.63) [12]. However, the causal relationship between AD and diabetes remains controversial [17–19], which makes it indispensable and significant to verify the relationship between AD and diabetes. In addition, due to residual confounding in observational studies with different ethnicities of the population as well as different sample sizes and data collection methods, there can be bias in the process of deciphering the relationship between AD and diabetes.

As a method in genetic epidemiology, Mendelian randomization (MR) is widely used for its practical and economic advantages. It involves using genetic variants of a disease as instrumental variables (IVs) to explore whether there is a causal relationship between exposure and outcomes [20, 21]. As genetic variants are randomly assigned at meiosis, MR studies are able to reduce the risk of confounding factors and to minimize the susceptibility of reverse causality [20, 21]. In this MR study, we analyzed the summary statistics to explore the causal relationship between AD and diabetes, thus providing new ideas for the management of diabetes. We present a Strengthening the Reporting of Observational Studies in Epidemiology (STROBE) for this MR study (Additional File 1) [22].

## Methods

### Study design

**Fig. 1:**
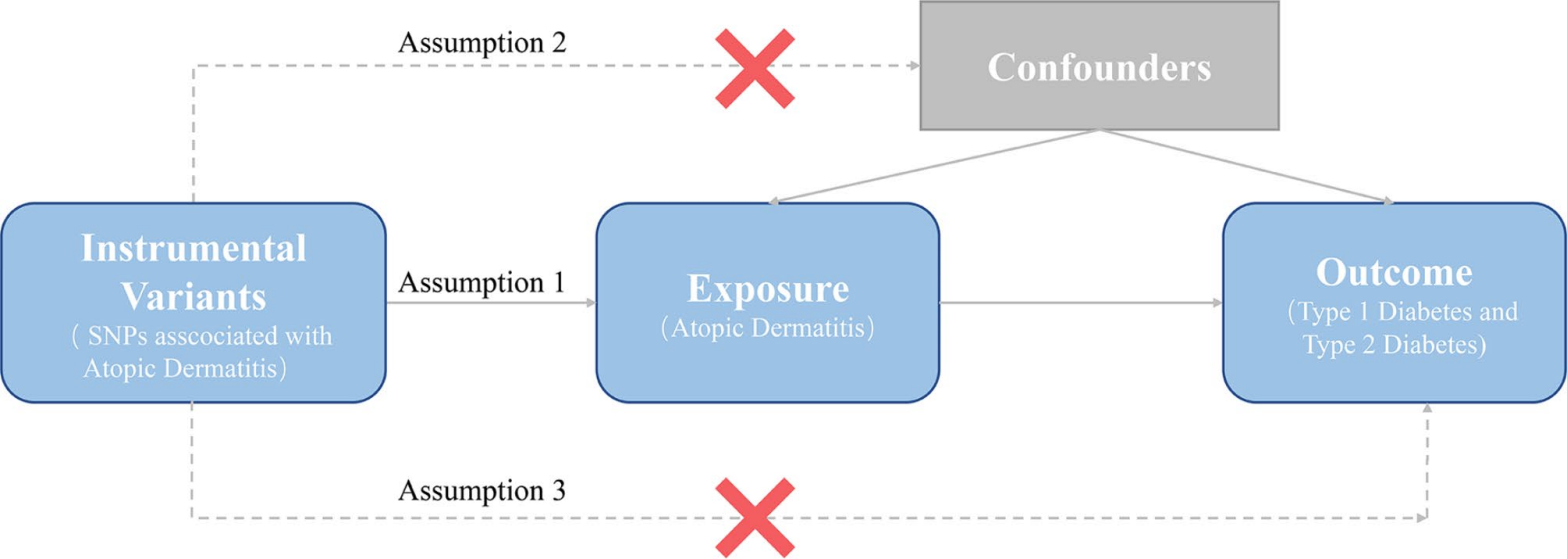
Study design flow diagram of Mendelian randomization (MR). Three key assumptions should be met: Assumption 1: Instrumental variables (IVs) should directly and significantly affect the risk of atopic dermatitis (AD). Assumption 2: IVs associated with any potential confounders should be absolutely avoided. Assumption 3: IVs should only affect type 1 diabetes (T1D) and type 2 diabetes (T2D) through AD. SNPs, single nucleotide polymorphisms

The process of this MR analysis is shown in Fig. 1. Overall, genetic variations were used as IVs to reveal the relationship between AD and both T1D and T2D based on three key hypotheses [23]. Firstly, IVs should directly and significantly affect the risk of AD. Secondly, IVs associated with any potential confounders should be absolutely avoided. Thirdly, IVs should only affect T1D and T2D through AD. Ethical approval and informed consent were obtained in the original studies.

### Data sources

Summary statistics and detailed data sources for T1D and T2D in this MR study are provided in Table 1. Summary-level statistics for T1D were derived from two recent large datasets: meta-analysis of genome-wide association study (GWAS) on T1D from Forgetta et al. (9,358 cases and 15,705 controls) [24] and FinnGen consortium (5,928 cases and 183,185 controls). Cases were defined by International Classification of Diseases (ICD)-8 in FinnGen and are available online at https://gwas.mrcieu.ac.uk/datasets/finn-b-E4_DM1/. Summary-level data for T2D were derived from a European-descent meta-analysis (74,124 cases, 824,006 controls) [25] based on 32 studies and another GWAS datasets from Xue et al., including 62,892 cases and 596,424 controls [26].

**Table 1:** Summary statistics and data sources in this MR study

Data Source	Phenotype	Sample Size	Cases	Population
EAGLE	AD	116,863	21,399	European
Forgetta et al.	T1D	25,063	9,358	European
FinnGen		189,113	5,928	European
Mahajan et al.	T2D	898,130	74,124	European
Xue et al.		659,316	62,892	European

EAGLE, EArly Genetics & Lifecourse Epidemiology; AD, atopic dermatitis; T1D, type 1 diabetes; T2D, type 2 diabetes

To investigate whether higher genetical levels of AD increased the odds of T1D and T2D, we selected single nucleotide polymorphisms (SNPs) as IVs for AD identified in a largest GWAS meta-analysis performed by the EArly Genetics & Lifecourse Epidemiology (EAGLE) eczema consortium (21,399 cases, 95,464 controls) [27]. All participants included in this study were of European ancestry. No samples overlap except between EAGLE and Mahajan et al. [25] (Additional File 2: Table S1).

### IVs selection

We extracted 21 SNPs with genome-wide significance (*P* < 5 × 10^− 8^) in the EAGLE study [27]. An SNP (rs12730935) was removed due to linkage disequilibrium (r^2^ < 0.01, clump distance < 10,000 kb) [28] based on 1000 genomes European population [29]. SNPs with minor allele frequencies (MAF) < 0.01 also need to be excluded since they usually tend to have low confidence and no SNPs were excluded in this step. To exclude those SNPs with potential confounders, we searched each of these in the PhenoScanner database [30] to satisfy the second fundamental assumption that IVs should avoid being associated with potential confounders. SNP rs4713555 was excluded because it was significantly associated with potential factors for T1D and T2D, including “Medication for cholesterol, blood pressure or diabetes: insulin” (*P* < 5 × 10^− 8^). To satisfy the third key assumption that IVs should affect T1D and T2D through AD only, we performed MR-Steiger analysis [31] and removed two SNPs “rs10214237 and rs6827756”, because they were demonstrated to explain more of the outcomes than AD and suggested a reverse causal relationship in Forgetta et al. (Additional File 2: Table S3) [24]. Since two SNPs (rs12188917 and rs6419573) could not be found in Forgetta et al. [24] and Mahajan et al. [25], three SNPs (rs12188917, rs6419573 and rs4809219) could not be found in and Xue et al. [26], we searched proxy-SNPs (r^2^ > 0.8) from an online website (http://snipa.helmholtz-muenchen.de/snipa3/) as a substitute (rs6596090 for rs12188917, rs1035127 for rs6419573, rs6011018 for rs4809219, respectively). Three SNPs were excluded (rs61813875, rs7127307, rs12951971) since they could neither be found nor replaced in Xue et al. [26].

We calculated the R^2^ and F statistic to assess the presence of weak IVs. It is generally accepted that the F statistics higher than the threshold of 10 indicates a low risk of weak IV bias. F = R^2^(N − 2)/(1 - R^2^) [32], where R^2^ indicates the degree of explanation of AD by IVs [33], N indicates the sample size. These 19 SNPs collectively explained 6.76% of the genetic variance of AD. All F statistics are higher than 10, indicating the absence of any weak IVs (Additional File 2: Table S2). Ultimately, we obtained 19 SNPs as IVs for this MR analysis, while only 17 SNPs for Forgetta et al. [24], 16 SNPs for Xue et al. [26]. (Additional File 2: Table S2 and 3).

### Statistical analysis

The associations of SNP-AD and SNP-diabetes were combined into one ratio to estimate causal effects. Inverse variance weighting (IVW) of different models was the predominant approach for this MR analysis [34], which provides the highest statistical power when the three key MR assumptions mentioned earlier are met and is more reliable for estimation when there is heterogeneity among SNPs. In addition, to make the results more reliable and robust, we performed a set of complementary analyses. Even if up to 50% of the information in the analysis comes from invalid IVs, the weighted median method still allows the results to be an unbiased estimate of causality [35]. Simple median method with equal weights was also used to estimate causality [35]. Due to its robustness in identifying pleiotropy, the MR-robust adjusted profile score (MR-Raps) was well received [36]. MR-PRESSO outlier test can detect outliers thus providing a more precisely MR estimation after removing them [37]. Scatter plots were provided to describe the causal relationship between genetically predicted AD and both T1D and T2D. Two-sided *P* < 0.05 was considered statistically significant. For multiple comparisons, Bonferroni correction was performed (*P* < 0.05/2).

Based on a type I error rate threshold of 0.05, power calculations were performed by calculating each study’s sample size, the proportion of cases and the explanation of variance by mRND [38].

### Sensitivity analyses

Heterogeneity among IVs was assessed by the calculation of Cochran’s Q and I^2^ statistics [39]. A Cochran’s Q *P* value of < 0.025 (0.05/2) or I^2^ statistic > 25% implies a heterogeneity that cannot be ignored [39], then IVW random-effects was considered to estimate the MR results with; otherwise, the IVW fixed-effects model was used [40]. MR-Egger regression was used to calculate the horizontal pleiotropy by estimating the intercept based on weighted linear regression of SNP-diabetes genetic susceptibility on SNP-AD associations [41]. A *P* value < 0.025 (0.05/2) of MR-Egger regression implies a potential bias in the IVW estimates. Meta-analysis combining the results of two T1D datasets and two T2D datasets respectively was performed by random-effects model without any significant heterogeneity (I^2^ = 0%, *P* = 0.50 for T1D; I^2^ = 0%, *P* = 0.82 for T2D) for the sake of estimating a more robust result. Leave-one-out method possesses powerful features to detect the bias of any single SNP on MR results [42]. SNPs strongly and independently influenced causality by leave-one-out method were retained.

R packages ‘TwoSampleMR’ [43], ‘MR-PRESSO’ [37] and ‘mr.raps’ [36] were used for this MR analysis. All statistical analyses for this study were performed in R software (version 4.1.3).

## Results

### Causal estimates between AD and the risk of diabetes

**Fig. 2:**
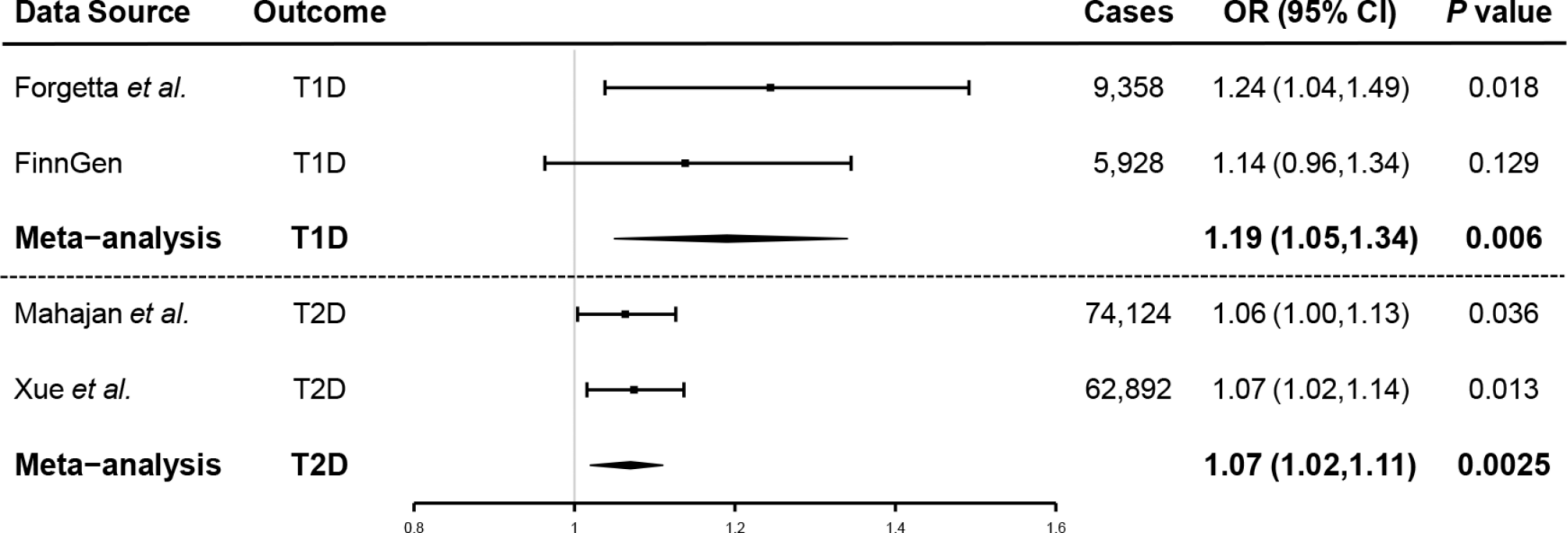
Association of genetically predicted AD on the risk of both T1D and T2D. Results were obtained from the inverse variance-weighted method in the random-effects model. AD, atopic dermatitis; T1D, type 1 diabetes; T2D, type 2 diabetes; OR, odds ratio; CI, confidence interval

**Fig. 3:**
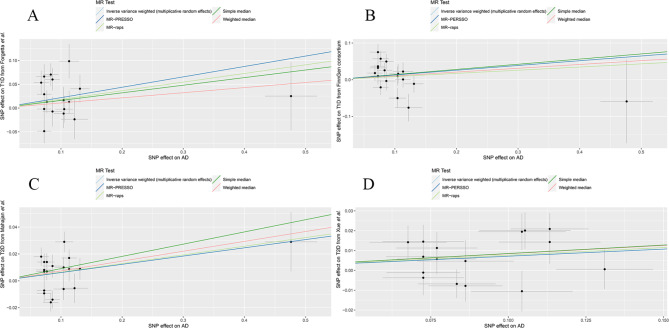
Scatter plot of the MR estimates for the association of AD with the risk of T1D and T2D based on Forgetta et al. **(A)**, FinnGen **(B)**, Mahajan et al. **(C)** and Xue et al. **(D)**. AD, atopic dermatitis; T1D, type 1 diabetes; T2D, type 2 diabetes; MR-Raps, MR Robust adjusted profile score; MR-PERSSO, MR Pleiotropy Residual Sum and Outlier

Main findings are presented in Fig. 2. The random-effects IVW analysis indicated that genetically predicted AD was positively associated with increased risk of T1D (OR, 1.24; 95% confidence interval (CI), 1.04, 1.49; *P* = 0.018) in Forgetta et al. Despite the null causal relationship found between AD and T1D in FinnGen consortium, meta-analysis by combining Forgetta et al. and FinnGen consortium reinforced the positive causal relationship (OR, 1.19; 95% CI, 1.05, 1.34; *P* = 0.006) **(**Fig. 2**).** We used the same approach to analyze the causal relationship between AD and T2D. Genetically predicted AD led to a higher risk of T2D in Xue et al. by random-effects IVW (OR, 1.07; 95% CI, 1.02, 1.14; *P* = 0.013). AD presented a suggestive significance for the risk of developing T2D in Mahajan et al. (OR, 1.06; 95% CI, 1.00, 1.13; *P* = 0.036). Meta-analysis by combining Xue et al. and Mahajan et al. consolidated the result (OR, 1.07; 95% CI, 1.02, 1.11; *P* = 0.003) **(**Fig. 2**).** Complementary analyses showed a consistent causal direction with the random-effects IVW analysis **(**Table 2; Fig. 3**)**.

**Table 2:** Association of genetically predicted AD with T1D and T2D risk in complementary analyses

Data Source	Outcome	SNPs, n	Methods	OR	95% CI	*P* value
Forgetta et al.	T1D	17	Weighted median	1.11	0.92, 1.35	0.276
		17	Simple median	1.17	0.96, 1.43	0.114
		17	MR-raps	1.20	1.00, 1.43	0.045
		17	MR-PRESSO^†^	NA	NA	NA
FinnGen	T1D	19	Weighted median	1.11	0.93, 1.33	0.255
		19	Simple median	1.15	0.96, 1.39	0.138
		19	MR-raps	1.09	0.91, 1.30	0.340
		19	MR-PRESSO^†^	NA	NA	NA
Mahajan et al.	T2D	19	Weighted median	1.08	1.02, 1.14	0.011
		19	Simple median	1.10	1.04, 1.16	0.001
		19	MR-raps	1.07	1.01, 1.12	0.014
		16	MR-PRESSO^‡^	1.08	1.03, 1.13	0.007
Xue et al.	T2D	16	Weighted median	1.09	1.01, 1.17	0.025
		16	Simple median	1.09	1.01, 1.17	0.023
		16	MR-Raps	1.08	1.02, 1.15	0.015
		16	MR-PRESSO^†^	NA	NA	NA

AD, atopic dermatitis; T1D, type 1 diabetes; T2D, type 2 diabetes; SNPs, single nucleotide polymorphisms; MR-Raps, MR-Robust adjusted profile score; MR − PRESSO, MR − pleiotropy residual sum and outlier

^†^No outliers detected

^‡^Calculated after removing 3 outlier SNPs (rs2212434, rs2041733, rs4809219)

Three SNPs with significant pleiotropy were identified by MR-PRESSO outliers test (rs2212434, rs2041733, rs4809219) in Mahajan et al. (Additional File 2: Table S4). After removing these outliers, we performed a replicate analysis (OR, 1.08; 95% CI, 1.03, 1.13; *P* = 0.002), and found that the results maintained the same direction without any horizontal pleiotropy by MR-Egger intercept (Additional File 2: Table S5).

### Sensitivity analyses of MR

**Fig. 4:**
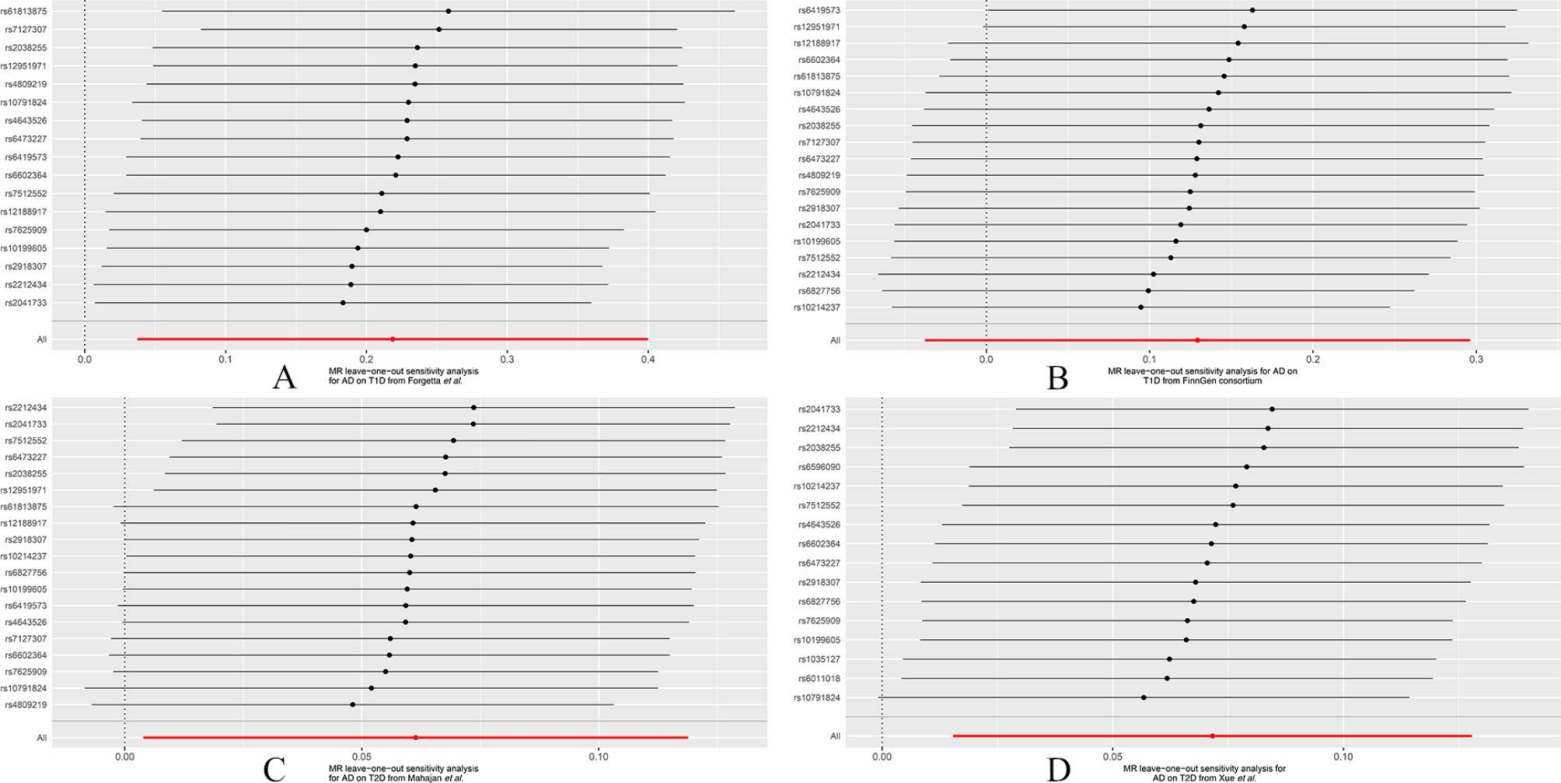
Leave-one-out plots for the MR analyses of AD on both T1D and T2D based on **(A)** Forgetta et al., **(B)** FinnGen, **(C)** Mahajan et al. and **(D)** Xue et al. Leave-one-out sensitivity analysis possesses powerful features to detect the bias of any single SNP on MR results. AD, atopic dermatitis; T1D, type 1 diabetes; T2D, type 2 diabetes; SNPs, single nucleotide polymorphisms

Cochran’s Q and I^2^ statistics indicated moderate heterogeneity between AD and both T1D and T2D (*P*_Cochran’s Q_ < 0.025 or I^2^ > 25%) **(**Table 3**).** However, we did not detect horizontal pleiotropy by MR-Egger Intercept p (threshold set at *P* < 0.025) except for FinnGen **(**Table 3, Additional File: Table S4). In leave-one-out analysis, several SNPs crossed the zero line after being removed (in Mahajan et al. and Xue et al.), while removing rs6419573 did not cause such a change (in FinnGen), indicating an individual SNP-driven causal estimation of genetically predicted AD on the risk of T1D and T2D. Attention needs to be directed to the robustness of causal relationships and result interpretations with caution **(**Fig. 4**).**

**Table 3:** Assessing heterogeneity and horizontal pleiotropy by different methods

Data Source	Outcome	Heterogeneity						Pleiotropy	
		IVW			MR-Egger			MR-Egger	
		Q/Q_df	Cochran’s Q *P*	I^2^ (%)	Q/Q_df	Cochran’s Q *P*	I^2^ (%)	Intercept	Intercept *P*
Forgetta et al.	T1D	31.53/16	0.012	49	29.26/15	0.015	49	0.025	0.297
FinnGen	T1D	37.85/18	0.004	52	27.55/17	0.051	38	0.061	0.022
Mahajan et al.	T2D	50.86/18	< 0.001	65	50.76/17	< 0.001	67	-0.001	0.859
Xue et al.	T2D	21.26/15	0.129	29	21.07/14	0.100	34	-0.005	0.73

Q, heterogeneity statistic Q; df, degree of freedom; I^2^ = (Q - Q_df ) / Q; AD, atopic dermatitis; T1D, type 1 diabetes; T2D, type 2 diabetes; IVW, the inverse variance weighting method; MR-Egger, Mendelian Randomization-Egger.

### Power calculations of MR

This study had a sufficient power (> 80%) to detect OR of 1.24 for T1D based on Forgetta et al. (Power = 99%), OR of 1.06 for T2D based on Mahajan et al. (Power = 98%), and OR of 1.07 for T2D based on Xue et al. (Power = 99%). However, it did not provide enough confidence (Power = 78%) to calculate the OR of 1.14 for T1D based on the FinnGen consortium.

## Discussion

To our knowledge, it is the first study to systematically explore the causal relationship between AD and diabetic risk by the approach of MR. We found that AD could increase the risk of both T1D and T2D in the European population.

AD is a chronic inflammatory skin disease primarily driven by T helper (Th) 2 and characterized by frequent episodes of persistent pruritus. Its growing prevalence causes a huge skin health burden and family financial burden both in the pediatric and adult populations [44]. The pathogenesis of AD is complex and usually involves the interaction between genetic susceptibility, skin barrier abnormalities, immune dysfunction and environmental factors [45]. Reduced expression of the protein filaggrin induced by mutations in *FLG* gene is found in 50% of AD patients [46, 47], which increases the risk of early-onset AD and is recognized as a major genetic predisposing factor for AD [48]. Lower levels of total ceramides accelerate water loss from the stratum corneum of skin among AD patients [49]. In the acute phase, AD is characterized by Th2 polarization [50, 51]. Pro-inflammatory cytokines induced by epidermal barrier damage activate innate immune components, leading to massive production of Th2 cytokines such as IL-4, IL-5 and IL-13 [52, 53].

As an autoimmune disease mediated primarily by Th1, T1D also displays association with AD by accumulating evidences. A large case-control study from Sweden including 104,832 AD cases and 1,022,435 controls showed that AD was significantly associated with multiple autoimmune disorders including T1D [15]. Wu et al. showed a higher prevalence of T1D in AD patients by analyzing 41,950 cases and 167,800 controls from the National Health Insurance Research Database (NHIRD) of Taiwan [13]. Several potential mechanisms have been proposed to explain these findings. As a Th2 cytokine, IL-4 contributes to autoimmune diabetes through increased expression of self-antigens in pancreatic islets [54]. Anderson et al. considered that β-cell destruction in T1D is a Th2-, not a Th1-mediated event [55]. Recent studies suggest that IL-4, IL-17 and IL-33 are simultaneously involved in the pathogenesis of AD and T1D by regulating autoimmune responses, implying the possibility of shared pathological process between AD and T1D [56]. As it is difficult to explain the association between T1D and AD by the traditional Th1/Th2 paradigm, an upgraded model with sophisticated T cell functional compartmentation may help to illustrate the underlying mechanism [57, 58].

Our findings in exploring the causal relationship between AD and T2D are consistent with several previous studies. Results from a National Health Interview Survey (NHIS) showed that AD increases the risk of lifetime prodromal diabetes [12]. Compared to controls, AD patients had a significantly higher risk of metabolic disorders such as hyperlipidemia and T2D [16]. Kok et al. found a significant association between moderate to severe AD and metabolic complications such as hypertension, hyperlipidemia, and T2D [59]. The exact mechanism how AD increases the risk of T2D is not clear. Chronic low-grade inflammation and immune system activation may function in the pathogenesis of obesity-related metabolic disorders [60–63]. T2D patients carry an elevated incidence of filaggrin null mutations, which is highly consistent with what happens in AD [64]. T2D patients expressed higher levels of IL-4 and IL-5 in their serum, suggesting the role of Th2-mediated inflammatory responses [65]. In addition, IL-17 exacerbated the inflammatory state in T2D, and IL-13 was significantly elevated in the serum of insulin-resistant patients [66, 67]. Based on above evidences, both AD and T2D have similar over-production of cytokines and inflammatory mediators, which may account for the increased risk of T2D in AD patients.

However, controversies still exist concerning the association between AD and diabetes. A population-based cohort study showed that AD did not increase the risk of T1D [68]. Schmitt et al. concluded that AD was associated with a reduced risk of T1D [18]. Andersen et al. showed adult AD patients either treated as inpatients or outpatients are unrelated to risk of new-onset T2D [69]. A cross-sectional study from Canada even showed that AD is a protective factor for T2D [70]. These inconsistent results could be attributed to several aspects of reasons. First, observational studies have their intrinsic limitations of selection and information biases that may lead to inaccurate results. Second, the conventional Th1/Th2 model may be insufficient to describe the immune dysregulation of T1D and AD. Third, environmental factors are involved in the occurrence of T2D, while MR study is based on the genetic level. Finally, MR study explains the lifetime effect of AD on diabetes, whereas observational studies are usually based on a limited period. Though we used the MR-Steiger method to exclude potential reverse causal confounding, it only supported a uni-directional causality and failed to explore the risk of AD in T1D patients. Therefore, a series of subsequent studies are still needed to investigate the relationship between AD and diabetes.

Generally, the IVW method provides the highest statistical power than other MR approaches in cases where the three key assumptions of MR are met without any significant pleiotropy among SNPs [34]. Most MR studies consider IVW as a primary analysis method [71], which becomes more persuasive after meta-analysis [72]. Several complementary analyses including weighted median, simple median and MR-raps between AD and T1D indicated a null causal association, which required us to pay more attention to the robustness of the results. However, all of complementary analyses provided consistent beta direction, which was strictly required by researchers in most MR studies [73, 74].

MR study is an emerging method utilizing genetic variations to explore the causal relationship between exposure and outcomes. The present study was designed upon the MR framework and showed distinct advantages. Environmental factors including diet, air pollutant and microbiota are involved in the onset of AD, which are inevitable confounders in observational studies and may contribute to the controversial causal relationship between AD and diabetes [75]. However, the instrumental variables consisting of SNPs overcame the causality effect limited to a span of time in observational studies, excluded the potential interference of residual confounders and were not affected by reverse causality with MR-Steiger analysis in current MR study, leading to more credible results and more convincing clinical guidance. All studies used for the analysis were based on European ancestry, thus avoiding causal bias due to ethnic differences.

The present study should be viewed in the light of its limitations. A nonlinear association between AD severity and diabetes could be neglected since our study was based on summary data. Whereas sensitivity analyses were performed, we need to be aware of the heterogeneity among SNPs. Horizontal pleiotropies were detected and the statistical power threshold of 80% of the association between AD and T1D from FinnGen was failed to reach. Several SNPs could drive the results separately and sample overlap existed between EAGLE and Mahajan et al. These factors may lead to bias in estimating causality and need our attention. MR-Egger was abandoned for use to estimate causality since the algorithm would lead to overly wide CIs, potentially leading to incorrect conclusions.

## Conclusion

As the first Mendelian randomization study to explore the causal association between AD and diabetes, the present study advocated that AD contributed to the occurrence of both T1D and T2D. These findings imply potential shared pathological processes underlying AD and diabetes and suggest that early prevention and diagnosis of AD may reduce the risk of developing T1D and T2D.

## Electronic supplementary material

Below is the link to the electronic supplementary material.

Supplementary Material 1

Supplementary Material 2

Supplementary Material 3

## Data Availability

The summary statistics of GWAS for atopic dermatitis are derived from a GWAS meta-analysis conducted by EAGLE consortium (https://www.eagle-consortium.org/); Full GWAS summary statistics for T1D are publicly available through FinnGen consortium (https://www.finngen.fi/en) and Forgetta et al. (Pubmed ID:32,005,708); Summary level data for T2D can be accessed from Mahajan et al. (Pubmed ID:30,297,969) and Xue et al. (Pubmed ID:30,054,458)

## Abbreviations

AD: Atopic dermatitis
T1D: Type 1 diabetes
T2D: Type 2 diabetes
MR: Mendelian randomization
OR: Odds ratio
HR: Hazard ratio
CI: Confidence interval
GWAS: Genome-wide association study
SNPs: Single nucleotide polymorphisms
IVs: Instrumental variables
IVW: Inverse variance weighted
CVD: Cardiovascular disease
MAF: Minor allele frequencies
MR-PRESSO: MR-pleiotropy residual sum and outlier
MR-Raps: MR-robust adjusted profile score
Th: T helper cell
