# Supplementary material for: Mendelian randomization indicates that atopic dermatitis contributes to the occurrence of diabetes
Source: BMC Med Genomics. 2023 Jun 15;16:132. doi: 10.1186/s12920-023-01575-y (PMC10268454; doi:10.1186/s12920-023-01575-y)
Supplement: Supplementary file 2 — Supplementary Material 2 [file 12920_2023_1575_MOESM2_ESM.docx]

**Table S1. Contributing studies of the datasets used for analyses.**

| Use in this MR | Phenotype | Data source | Contributing studies | Sample overlap |
| --- | --- | --- | --- | --- |
| Exposure | Atopic Dermatitis | EAGLE | 23andMe_V2, 23andMe_V3, AAGC, ALSPAC, B58C, BAMSE, CHOP, COPSAC2000, DNBC, GALA II, GenerationR-CAUC, GenerationR-NONCAUC, GENEVA/KORA F4/POPGEN, GENUFAD-SHIP2, GENUFADex-SHIP1, GINI/LISA, INMA, MAAS, MAS-HNR, MoBa, NCRC-ADC, NFBC66, NTR, RIKEN, SAPALDIA, SAPPHIRE. | / |
| Outcomes | Type 1 Diabetes | Forgetta *et al.* | Affymetrix 500K array, GoKinD, WTCCC, HumanHap550 or Human1-2M-DuoCustom_v1_A, McGill University, CHOPTDT, DCCT-EDIC, T1DGC, TwinsUK, 58BC, NBS, UK Biobank. | 0 |
|  | Type 1 Diabetes | Finngen | Not Reported. | 0 |
|  | Type 2 Diabetes | Mahajan *et al.* | DANISH-UCPH, EGCUT, FHS, FUSION, GCKD, GENOA, GERA, GoDARTS, GoMAP and TEENAGE, InterAct, KORA, MESA, METSIM, MGI, Mount Sinai BioMe Biobank, PIVUS and ULSAM, PROSPER, The Rotterdam Study, WTCCC, UK Biobank. | KORA |
|  | Type 2 Diabetes | Xue *et al.* | DIAGRAM, GERA, UK Biobank. | 0 |

EAGLE, EArly Genetics & Lifecourse Epidemiology; DIAGRAM, DIAbetes Genetics Replication and Meta-analysis;GERA, Genetic Epidemiology Research on Aging.

**Table S2. Characteristics of the genetic variants associated with atopic dermatitis.**

| SNP | EA | OA | N | EAF | R²(%) | F | OR | SNP-Atopic dermatitis | | | SNP-T1D (Forgetta *et al.*) | | | SNP-T1D (FinnGen) | | | SNP-T2D (Mahajan *et al.*) | | | SNP-T2D (Xue *et al.*) | | |
| --- | --- | --- | --- | --- | --- | --- | --- | --- | --- | --- | --- | --- | --- | --- | --- | --- | --- | --- | --- | --- | --- | --- |
|  |  |  |  |  |  |  |  | *P* value | Beta | SE | *P* value | Beta | SE | *P* value | Beta | SE | *P* value | Beta | SE | *P* value | Beta | SE |
| rs61813875¢ | G | C | 93,326 | 0.02 | 0.888 | 836 | 1.61 | 5.60E-29 | 0.476 | 0.043 | 7.25E-01 | 0.025 | 0.072 | 6.04E-01 | -0.059 | 0.114 | 1.80E-01 | 0.029 | 0.022 | NA | NA | NA |
| rs10791824 | G | A | 102,761 | 0.57 | 0.626 | 647 | 1.12 | 2.10E-19 | 0.113 | 0.013 | 5.89E-01 | 0.013 | 0.024 | 9.76E-01 | 0.001 | 0.022 | 8.70E-03 | 0.017 | 0.006 | 8.64E-03 | 0.113 | 0.008 |
| rs12188917* | C | T | 102,761 | 0.21 | 0.569 | 588 | 1.14 | 4.00E-17 | 0.131 | 0.016 | 1.67E-01 | 0.041 | 0.03 | 6.41E-01 | -0.011 | 0.024 | 2.60E-01 | 0.009 | 0.008 | 9.48E-01 | 0.131 | 0.01 |
| rs6419573* | T | C | 102,760 | 0.26 | 0.416 | 429 | 1.11 | 1.50E-13 | 0.104 | 0.014 | 5.49E-01 | 0.017 | 0.028 | 6.18E-02 | -0.05 | 0.027 | 1.90E-01 | 0.01 | 0.008 | 3.46E-02 | 0.104 | 0.009 |
| rs2212434 | T | C | 102,761 | 0.45 | 0.366 | 378 | 1.09 | 4.60E-13 | 0.086 | 0.012 | 1.04E-02 | 0.06 | 0.024 | 1.93E-02 | 0.05 | 0.021 | 3.60E-02 | -0.014 | 0.007 | 3.21E-01 | 0.086 | 0.008 |
| rs4809219∂ | C | A | 102,760 | 0.27 | 0.435 | 449 | 0.9 | 7.00E-13 | -0.105 | 0.015 | 9.33E-01 | 0.002 | 0.028 | 5.42E-01 | -0.016 | 0.026 | 1.60E-04 | -0.029 | 0.008 | 2.86E-02 | -0.105 | 0.009 |
| rs2918307 | G | A | 100,707 | 0.16 | 0.343 | 347 | 1.12 | 4.60E-12 | 0.113 | 0.016 | 6.48E-03 | 0.099 | 0.036 | 3.76E-01 | 0.022 | 0.025 | 3.30E-01 | 0.009 | 0.009 | 1.90E-01 | 0.113 | 0.011 |
| rs2041733 | C | T | 103,066 | 0.55 | 0.341 | 353 | 0.92 | 2.50E-11 | -0.083 | 0.012 | 3.10E-03 | -0.07 | 0.024 | 2.21E-01 | -0.026 | 0.021 | 1.90E-02 | 0.016 | 0.007 | 3.42E-01 | -0.083 | 0.007 |
| rs6827756º | T | C | 102,761 | 0.37 | 0.276 | 285 | 1.08 | 4.20E-09 | 0.077 | 0.013 | 2.00E-06 | 0.115 | 0.024 | 6.24E-03 | 0.057 | 0.021 | 2.90E-01 | 0.007 | 0.007 | 1.68E-01 | 0.077 | 0.008 |
| rs2038255 | T | C | 102,760 | 0.18 | 0.319 | 329 | 1.11 | 1.80E-10 | 0.104 | 0.016 | 7.06E-01 | -0.012 | 0.031 | 7.02E-01 | 0.01 | 0.026 | 4.50E-01 | -0.006 | 0.008 | 3.01E-01 | 0.104 | 0.01 |
| rs7127307¢ | C | T | 103,066 | 0.47 | 0.265 | 274 | 0.93 | 3.90E-10 | -0.073 | 0.012 | 5.45E-02 | 0.049 | 0.025 | 7.05E-01 | -0.008 | 0.021 | 3.30E-02 | -0.014 | 0.006 | NA | NA | NA |
| rs7512552 | T | C | 102,762 | 0.49 | 0.266 | 274 | 0.93 | 9.10E-10 | -0.073 | 0.012 | 2.19E-01 | -0.029 | 0.024 | 7.97E-02 | -0.037 | 0.021 | 1.40E-01 | 0.009 | 0.006 | 8.89E-01 | -0.073 | 0.008 |
| rs6473227 | A | C | 102,761 | 0.61 | 0.254 | 261 | 0.93 | 1.40E-09 | -0.073 | 0.012 | 9.34E-01 | 0.002 | 0.024 | 6.43E-01 | -0.01 | 0.022 | 2.70E-01 | 0.007 | 0.007 | 3.96E-01 | -0.073 | 0.008 |
| rs6602364 | G | C | 103,065 | 0.45 | 0.293 | 303 | 1.08 | 1.50E-09 | 0.077 | 0.013 | 5.80E-01 | 0.013 | 0.024 | 3.25E-01 | -0.021 | 0.021 | 3.00E-02 | 0.014 | 0.006 | 4.57E-01 | 0.077 | 0.008 |
| rs10214237º | C | T | 102,761 | 0.27 | 0.21 | 216 | 0.93 | 2.90E-08 | -0.073 | 0.013 | 2.81E-06 | -0.125 | 0.027 | 7.06E-04 | -0.075 | 0.022 | 3.40E-01 | -0.007 | 0.007 | 6.73E-01 | -0.073 | 0.009 |
| rs10199605 | A | G | 102,760 | 0.3 | 0.224 | 231 | 0.93 | 3.40E-08 | -0.073 | 0.013 | 1.02E-02 | -0.066 | 0.026 | 1.28E-01 | -0.032 | 0.021 | 2.40E-01 | -0.008 | 0.007 | 8.98E-02 | -0.073 | 0.009 |
| rs4643526 | A | G | 103,066 | 0.19 | 0.228 | 235 | 1.09 | 3.50E-08 | 0.086 | 0.016 | 8.19E-01 | -0.007 | 0.031 | 8.90E-01 | -0.004 | 0.025 | 1.80E-01 | 0.011 | 0.009 | 6.52E-01 | 0.086 | 0.011 |
| rs12951971¢ | G | T | 102,761 | 0.09 | 0.244 | 251 | 1.13 | 4.10E-08 | 0.122 | 0.022 | 5.68E-01 | -0.024 | 0.042 | 4.26E-02 | -0.076 | 0.038 | 6.10E-01 | -0.006 | 0.011 | NA | NA | NA |
| rs7625909 | T | C | 102,761 | 0.32 | 0.201 | 207 | 1.07 | 4.90E-08 | 0.068 | 0.012 | 3.32E-02 | 0.053 | 0.025 | 4.19E-01 | 0.018 | 0.022 | 7.20E-03 | 0.018 | 0.007 | 9.22E-02 | 0.068 | 0.008 |
| rs4713555† | T | G | 91,217 | 0.27 |  |  |  |  |  |  |  |  |  |  |  |  |  |  |  |  |  |  |
| rs12730935‡ | A | G | 102,760 | 0.39 |  |  |  |  |  |  |  |  |  |  |  |  |  |  |  |  |  |  |

SNP, single-nucleotide polymorphism; EA, effect allele; OA, other allele; N, sample size; EAF, effect allele frequency; R2, percentage of the variation of AD explained by the SNPs; F, F statistic; Beta, the per-allele effect on AD; SE, standard error; P value is for the genetic association.

*rs12188917 and rs6419573 were not available in Forgetta *et al.*, Mahajan *et al*., and Xue *et al*.; rs6596090 and rs1035127 were found to replace them, respectively.

∂rs4809219 was not available in Xue *et al*. either, rs6011018 was found to replace it.

†rs4713555 was excluded since it was associated with potential confounders on T1D and T2D in the PhenoScanner database.

‡rs12730935 was excluded during linkage disequilibrium.

ºrs6827756 and rs10214237 were excluded since they were demonstrated to explain more of the outcomes than AD in Forgetta *et al*.

¢rs61813875, rs7127307, rs12951971 were excluded since they could neither be found nor replaced in Xue *et al*.

**Table S3. Results of MR Steiger direction test.**

| **Data source** | **Outcome** | **SNP** | **rsq.exposure** | **rsq.outcome** | **steiger_dir^*^** | **steiger_pval** |
| --- | --- | --- | --- | --- | --- | --- |
| Forgetta *et al.* | T1D | 1:150265704 | 3.65E-04 | 6.02E-05 | TRUE | 1.07E-01 |
|  |  | 1:152536650 | 1.34E-03 | 4.92E-06 | TRUE | 1.38E-06 |
|  |  | 10:6038853 | 3.54E-04 | 1.22E-05 | TRUE | 2.94E-02 |
|  |  | 11:128187383 | 3.80E-04 | 1.47E-04 | TRUE | 2.96E-01 |
|  |  | 11:65559266 | 7.89E-04 | 1.15E-05 | TRUE | 4.55E-04 |
|  |  | 11:76281593 | 5.09E-04 | 2.61E-04 | TRUE | 3.62E-01 |
|  |  | 14:35559126 | 3.96E-04 | 5.73E-06 | TRUE | 1.30E-02 |
|  |  | 16:11229589 | 4.32E-04 | 3.49E-04 | TRUE | 7.65E-01 |
|  |  | 17:40528131 | 2.93E-04 | 1.30E-05 | TRUE | 5.51E-02 |
|  |  | 19:8789722 | 4.75E-04 | 2.95E-04 | TRUE | 5.14E-01 |
|  |  | 2:103019919 | 5.31E-04 | 1.43E-05 | TRUE | 6.26E-03 |
|  |  | 2:61184651 | 2.95E-04 | 2.10E-06 | TRUE | 2.55E-02 |
|  |  | 2:8495097 | 2.96E-04 | 2.63E-04 | TRUE | 8.89E-01 |
|  |  | 20:62303115 | 5.01E-04 | 2.71E-07 | TRUE | 1.90E-03 |
|  |  | 3:53091164 | 2.89E-04 | 1.81E-04 | TRUE | 6.12E-01 |
|  |  | 4:123184411† | 3.36E-04 | 8.97E-04 | †FALSE | 9.87E-02 |
|  |  | 5:131988415 | 6.88E-04 | 7.59E-05 | TRUE | 1.29E-02 |
|  |  | 5:35883734† | 2.99E-04 | 8.71E-04 | †FALSE | 8.27E-02 |
|  |  | 8:81285892 | 3.57E-04 | 2.77E-07 | TRUE | 9.15E-03 |
| FinnGen | T1D | rs10199605 | 2.96E-04 | 1.22E-05 | TRUE | 3.98E-04 |
|  |  | rs10214237 | 2.99E-04 | 6.05E-05 | TRUE | 1.40E-02 |
|  |  | rs10791824 | 7.89E-04 | 5.50E-09 | TRUE | 4.80E-13 |
|  |  | rs12188917 | 6.88E-04 | 1.16E-06 | TRUE | 8.41E-11 |
|  |  | rs12951971 | 2.93E-04 | 2.18E-05 | TRUE | 1.32E-03 |
|  |  | rs2038255 | 3.96E-04 | 7.82E-07 | TRUE | 9.35E-07 |
|  |  | rs2041733 | 4.32E-04 | 7.86E-06 | TRUE | 3.41E-06 |
|  |  | rs2212434 | 5.09E-04 | 2.89E-05 | TRUE | 9.15E-06 |
|  |  | rs2918307 | 4.75E-04 | 4.14E-06 | TRUE | 4.07E-07 |
|  |  | rs4643526 | 2.95E-04 | 1.00E-07 | TRUE | 1.33E-05 |
|  |  | rs4809219 | 5.01E-04 | 1.97E-06 | TRUE | 6.07E-08 |
|  |  | rs61813875 | 1.34E-03 | 1.43E-06 | TRUE | 9.48E-19 |
|  |  | rs6419573 | 5.31E-04 | 1.85E-05 | TRUE | 1.32E-06 |
|  |  | rs6473227 | 3.57E-04 | 1.14E-06 | TRUE | 4.25E-06 |
|  |  | rs6602364 | 3.54E-04 | 5.14E-06 | TRUE | 1.89E-05 |
|  |  | rs6827756 | 3.36E-04 | 3.96E-05 | TRUE | 1.90E-03 |
|  |  | rs7127307 | 3.80E-04 | 7.60E-07 | TRUE | 1.52E-06 |
|  |  | rs7512552 | 3.65E-04 | 1.63E-05 | TRUE | 1.01E-04 |
|  |  | rs7625909 | 2.89E-04 | 3.45E-06 | TRUE | 9.18E-05 |
| Mahajan *et al.* | T2D | 1:150265704 | 3.65E-04 | 2.43E-06 | TRUE | 9.92E-08 |
|  |  | 1:152536650 | 1.34E-03 | 1.93E-06 | TRUE | 1.51E-24 |
|  |  | 10:6038853 | 3.54E-04 | 5.33E-06 | TRUE | 5.10E-07 |
|  |  | 11:128187383 | 3.80E-04 | 5.33E-06 | TRUE | 1.74E-07 |
|  |  | 11:65559266 | 7.89E-04 | 7.86E-06 | TRUE | 1.58E-14 |
|  |  | 11:76281593 | 5.09E-04 | 5.01E-06 | TRUE | 6.62E-10 |
|  |  | 14:35559126 | 3.96E-04 | 6.16E-07 | TRUE | 6.53E-09 |
|  |  | 16:11229589 | 4.32E-04 | 6.16E-06 | TRUE | 2.62E-08 |
|  |  | 17:40528131 | 2.93E-04 | 2.78E-07 | TRUE | 4.74E-07 |
|  |  | 19:8789722 | 4.75E-04 | 1.06E-06 | TRUE | 4.14E-10 |
|  |  | 2:103019919 | 5.31E-04 | 1.93E-06 | TRUE | 4.85E-11 |
|  |  | 2:61184651 | 2.95E-04 | 1.86E-06 | TRUE | 1.53E-06 |
|  |  | 2:8495097 | 2.96E-04 | 1.58E-06 | TRUE | 1.26E-06 |
|  |  | 20:62303115 | 5.01E-04 | 1.62E-05 | TRUE | 2.44E-08 |
|  |  | 3:53091164 | 2.89E-04 | 7.80E-06 | TRUE | 1.57E-05 |
|  |  | 4:123184411 | 3.36E-04 | 1.25E-06 | TRUE | 1.73E-07 |
|  |  | 5:131988415 | 6.88E-04 | 1.38E-06 | TRUE | 2.70E-14 |
|  |  | 5:35883734 | 2.99E-04 | 1.02E-06 | TRUE | 7.51E-07 |
|  |  | 8:81285892 | 3.57E-04 | 1.37E-06 | TRUE | 7.41E-08 |
| Xue *et al.* | T2D | rs10199605 | 2.96E-04 | 4.41E-06 | TRUE | 6.57E-06 |
|  |  | rs10214237 | 2.99E-04 | 2.68E-07 | TRUE | 5.58E-07 |
|  |  | rs1035127 | 5.31E-04 | 6.81E-06 | TRUE | 1.11E-09 |
|  |  | rs10791824 | 7.89E-04 | 1.04E-05 | TRUE | 1.19E-13 |
|  |  | rs2038255 | 3.96E-04 | 1.61E-06 | TRUE | 2.80E-08 |
|  |  | rs2041733 | 4.32E-04 | 1.35E-06 | TRUE | 4.68E-09 |
|  |  | rs2212434 | 5.09E-04 | 1.48E-06 | TRUE | 1.92E-10 |
|  |  | rs2918307 | 4.75E-04 | 2.61E-06 | TRUE | 2.45E-09 |
|  |  | rs4643526 | 2.95E-04 | 3.04E-07 | TRUE | 6.93E-07 |
|  |  | rs6011018 | 5.01E-04 | 7.24E-06 | TRUE | 4.23E-09 |
|  |  | rs6473227 | 3.57E-04 | 1.10E-06 | TRUE | 1.04E-07 |
|  |  | rs6596090 | 6.88E-04 | 5.46E-09 | TRUE | 6.08E-15 |
|  |  | rs6602364 | 3.54E-04 | 8.39E-07 | TRUE | 8.93E-08 |
|  |  | rs6827756 | 3.36E-04 | 2.88E-06 | TRUE | 7.08E-07 |
|  |  | rs7512552 | 3.65E-04 | 2.94E-08 | TRUE | 1.65E-08 |
|  |  | rs7625909 | 2.89E-04 | 4.33E-06 | TRUE | 8.48E-06 |

*SNPs with “TRUE” MR Steiger results suggest causality in the expected direction and will not be removed from the study.

†SNPs with “FALSE” MR Steiger results suggest causality in the reverse direction and will be removed from the study.

**Table S4. Evaluation of heterogeneity and directional pleiotropy using different methods.**

| Outcome | SNPs, n | Cochran’s Q  statistic | Cochran’s Q  *P* | MR-Egger intercept *P* | MR-PRESSO Outlier Test |
| --- | --- | --- | --- | --- | --- |
|  |  |  |  |  |  |
| Forgetta *et al.* | 17^*^ | 31.530 | 0.011 | 0.297 | ^†^ |
| FinnGen | 19 | 37.850 | 0.004 | 0.022 | ^†^ |
| Mahajan *et al.* | 19^*^ | 50.860 | < 0.001 | 0.859 | *†*rs2212434, rs2041733, rs4809219 |
| Xue A *et al.* | 16^*^ | 21.260 | 0.129 | 0.730 | ^†^ |

SNP, single-nucleotide polymorphism; MR-Egger, Mendelian randomization-Egger; MR-PRESSO, MR-pleiotropy residual sum and outlier.

*rs12188917 and rs6419573 were not available in Forgetta *et al*., Mahajan *et al*., and Xue *et al*.; rs6596090 and rs1035127 were found to replace them, respectively.

∂rs4809219 was not available in Xue *et al* either., rs6011018 was found to replace it.

ºrs6827756 and rs10214237 were excluded since they were demonstrated to explain more of the outcomes than AD in Forgetta *et al*.

¢rs61813875, rs7127307, rs12951971 were excluded since they could neither be found nor replaced in Xue *et al*.

†No outliers detected.

†Three outliers were detected in Mahajan *et al*. (*P* < 0.05)

**Table S5. Associations of genetic predisposition to AD with risk of T2D in Mahajan *et al*. after excluding 3 outliers (rs2212434, rs2041733, rs4809219).**

| Analyses | | SNPs, n | Methods | OR (95% CI) | *P* value |
| --- | --- | --- | --- | --- | --- |
| Results | | 16^*,#^ | random-effects IVW | 1.08 (1.03, 1.13) | 0.002 |
|  |  | 16^*,#^ | Weighted median | 1.08 (1.02, 1.14) | 0.006 |
|  |  | 16^*,#^ | Simple median | 1.10 (1.04, 1.16) | 0.001 |
|  |  | 16^*,#^ | MR-raps | 1.08 (1.03, 1.13) | 0.001 |
|  |  | 16^*,#^ | MR-PRESSO† | 1.08 (1.03, 1.13) | 0.007 |
| Sensitivity Analyses | Heterogeneity test | 16^*,#^ | MR-Egger | / | 0.058 |
|  |  | 16^*,#^ | Inverse-variance weighted | / | 0.075 |
|  | Pleiotropy test | 16^*,#^ | MR-Egger | / | 0.692 |

| T2D, type 2 diabetes; SNPs, single-nucleotide polymorphisms; OR, odds ratio; CI, confidence interval. |
| --- |
| ^*^rs12188917 and rs6419573 were not available in Mahajan *et al.*; rs6596090 and rs1035127 were found to replace them, respectively. |
| ^#^rs4713555 and rs12730935 was excluded. |
