# Supplementary material for: Mendelian randomization indicates that atopic dermatitis contributes to the occurrence of diabetes
Source: BMC Med Genomics. 2023 Jun 15;16:132. doi: 10.1186/s12920-023-01575-y (PMC10268454; doi:10.1186/s12920-023-01575-y)
Supplement: Supplementary file 3 — Supplementary Material 3 [file 12920_2023_1575_MOESM3_ESM.docx]

**Additional File**

Additional File 1, format: .docx; Title & Description: STROBE-MR checklist of recommended items to address in reports of Mendelian randomization studies.

Additional File 2, format: .docx; Title & Description: Table S1: Contributing studies of the datasets used for analyses. Table S2: Characteristics of the genetic variants associated with atopic dermatitis. Table S3: Results of MR Steiger direction test. Table S4: Evaluation of heterogeneity and directional pleiotropy using different methods. Table S5: Associations of genetic predisposition to AD with risk of T2D in Mahajan *et al.* after excluding 3 outliers (rs2212434, rs2041733, rs4809219).
